# Maintaining a Twitter Feed to Advance an Internal Medicine Residency Program’s Educational Mission

**DOI:** 10.2196/mededu.4434

**Published:** 2015-07-10

**Authors:** Paul A Bergl, Akhil Narang, Vineet M Arora

**Affiliations:** ^1^ General Internal Medicine Department of Medicine Medical College of Wisconsin Milwaukee, WI United States; ^2^ Fellow in Cardiology Department of Medicine University of Chicago Chicago, IL United States; ^3^ Section of General Internal Medicine Department of Medicine University of Chicago Chicago, IL United States

**Keywords:** social media, medical education, Twitter messaging, Internet/ethics

## Abstract

**Background:**

Residency programs face many challenges in educating learners. The millennial generation’s learning preferences also force us to reconsider how to reach physicians in training. Social media is emerging as a viable tool for advancing curricula in graduate medical education.

**Objective:**

The authors sought to understand how social media enhances a residency program’s educational mission.

**Methods:**

While chief residents in the 2013-2014 academic year, two of the authors (PB, AN) maintained a Twitter feed for their academic internal medicine residency program. Participants included the chief residents and categorical internal medicine house staff.

**Results:**

At the year’s end, the authors surveyed residents about uses and attitudes toward this initiative. Residents generally found the chief residents’ tweets informative, and most residents (42/61, 69%) agreed that Twitter enhanced their overall education in residency.

**Conclusions:**

Data from this single-site intervention corroborate that Twitter can strengthen a residency program’s educational mission. The program’s robust following on Twitter outside of the home program also suggests a need for wider adoption of social media in graduate medical education. Improved use of data analytics and dissemination of these practices to other programs would lend additional insight into social media’s role in improving residents’ educational experiences.

## Introduction

The current learning environment for internal medicine residency contains potential obstacles to learning: duty hour restrictions, training at multiple sites, increasingly complex patients, and pressures for clinical productivity and timely discharge. Moreover, our trainees have evolved; the so-called millennial generation may require more interactive learning experiences and may favor technology-driven learning platforms [1]. The vast majority of millennials use social media personally and professionally, often accessing sites multiple times daily [2]. As residents face increasing competition for their attention, residency programs need to reconsider how they deliver educational offerings and reach trainees. Social media offers a novel approach to keep contemporary residents informed, to disseminate teaching materials, and to interact with learners.

We tested the use of a novel social media initiative—specifically, a Twitter feed—to enhance the education of our residents and the mission of our internal medicine residency program. A recent systematic review explored the role of social media in medical education; results of social media initiatives were largely mixed [3]. These initiatives resulted in increased feedback to learners and more learner satisfaction, but had equivocal effects on knowledge promotion [3]. The majority of these studies examined medical students; thus, few compelling data exist on the use of social media as a teaching tool in residency education, making this area ripe for testing and evaluation.

To our knowledge, no residency program has reported on its experience or outcomes in using social media to advance the program’s educational mission. Herein we describe our implementation strategy for using Twitter and report on its reception among residents. We specifically sought to understand Twitter’s value as an extension of our teaching conferences and as a more general educational resource for residents.

## Methods

### Overview

We conducted our educational intervention in a single internal medicine residency program at an academic medical center over one academic year. The intervention was led by the chief residents, and residents voluntarily participated on Twitter at their convenience.

Our program includes 95 categorical residents. During the intervention, our residents rotated through three hospitals on two campuses. This project represents a pilot curricular innovation. Regarding ethical approval, we have obtained Institutional Review Board (IRB) exemption for this study.

### Logistics

We selected Twitter for our platform based on its efficiency, propensity for publicity, its rapid growth, and its novelty. First, a well-curated Twitter feed can offer succinct, timely educational material for busy residents, including program-specific announcements, educational materials, and discussion points on the latest medical headlines. Next, because websites are static and require active maintenance, they do not promote learner engagement or the interactivity that Twitter offers. Finally, a Twitter feed improves the visibility of a program’s academic activities, including publicly highlighting the quality and diversity of teaching conferences. We could also promote residents’ publications, research posters, teaching awards, and community service work. This publicity may also represent a powerful recruitment tool. Table 1 shows a brief glossary of Twitter terminology.

We launched our @Medchiefs Twitter account in July 2013 while two of the authors (PB, AN) served as chief residents and the third author (VA) served as associate program director for the University of Chicago Internal Medicine Residency Program. Only the chief residents had the account’s password. We linked our Twitter account to Facebook, and thus all of our tweets were also simultaneously broadcasted to our followers’ Facebook feeds. We also embedded the Twitter feed directly into our chief residents’ website [4]. Because our website hosts our conference calendar, resident team schedules, vital administrative policies, and service rules, most residents access the site at least daily.

**Table 1:** Brief glossary of Twitter terminology.

Term	Meaning
@Username	Designation of a Twitter user; usernames always preceded by an @ sign
Tweet	*Noun:* A message consisting of 140 characters that is publicly displayed on the user's timeline *Verb:* To post a brief message on one's own timeline
Retweet	*Verb:* To repost another user's message verbatim to one's own timeline
Timeline	The running feed of messages posted or retweeted
Followers	The group of other users who has voluntary elected to follow one's own timeline
Hashtag	Designation of an indexed keyword; a searchable term or topic on Twitter

### Time Invested in Twitter

We estimate that the chief residents dedicated 30 to 60 minutes daily to actively maintaining our feed. This time included reviewing other users’ posts, messaging followers, and queueing up tweets for later dissemination. Additionally, we often tweeted in real time during conferences, such as weekly Department of Medicine Grand Rounds.

### Establishing or Using Hashtags

By searching specific “hashtags,” Twitter users can quickly find tweets or users within specific areas of interest. When possible, we applied hashtags to conference tweets such as #grandrounds. After reaching out to other programs on Twitter and reviewing hashtag trends, we settled on the nascent #AMReport to designate tweets from our internal medicine morning report. We also created a program-specific hashtag, #UCIMR, to encourage our residents to reference our program online.

### Types of Tweets and Their Purposes

We loosely adhered to our own “rule of thirds” when tweeting; a balanced number of tweets pertained to resident education, recognition of our program’s academic activities, and outward promotion.

Tweets about our morning report constituted the greatest number of indexed tweets; a total of 31% of our tweets related to the morning report conference. Tweets were used to broadcast teaching points from our morning report and summarize key features of the case.

In addition, tweets from teaching conferences linked to relevant medical literature discussed, and occasionally shared, educational radiographs, computerized tomography (CT) scans, or pathology slides from interesting cases. Any patient-related materials that were shared were stripped of all identifiers to avoid Health Insurance Portability and Accountability Act (HIPAA) violations.

Because resident-prepared morning report cases were reviewed by chief residents in advance, “tweetable” teaching points were prescheduled for broadcasting during the conference via Hootsuite, a third-party social media application. Using this approach prevented chief residents from being distracted during the conference and also offered the opportunity to reflect on the most valuable teaching points for each case.

We also tweeted relevant news for our residents by discriminating which medical headlines would apply most to our residents. In addition, we highlighted institution-specific accomplishments and examples of our faculty physicians being recognized in national and global forums.

### Advancing House Officer Recruitment

We recognized that our Twitter feed might also buttress our recruitment efforts. By having applicants follow us during the recruitment season, we could continually highlight our robust academic activities and dedication to resident education. As such, during the 2013-2014 interview season, we distributed flyers promoting our Twitter feed to all applicants on their interview day.

### Survey of Residents

At the end of the 2013-2014 academic year, we conducted a brief 14-item survey on our residents’ use patterns with our Twitter account. Our survey asked residents how frequently they viewed the Twitter feed, how informative they found tweets, and how valuable they found the intervention. Our IRB determined this survey-based study to be exempt.

### Online Audience

Both Twitter and third-party companies Hootsuite and Simply Measured provide a number of metrics on followers and online activities. These metrics include online influence of followers, approximate locations of followers, and popularity of individual tweets. This online data was retrieved on July 23, 2014, approximately one year after the inception of our account.

## Results

In our first year, we amassed a following of more than 1000 Twitter users; we grew our global following by 3 users per day. At the end of the 2013-2014 academic year, we identified at least 27.1% (35/129) of our current residents, preliminary interns, and chief residents among our Twitter followers. We also published over 1000 tweets, or about 3 tweets per day. Our messages generated a total of 782 retweets.

The largest segment of our followers consists of health professionals as evidenced by the four most common keywords in our followers’ profiles: *medicine*, *medical*, *health*, and *physician*. Only 35% of our 1091 followers access Twitter from our program’s time zone; this data suggests that most our followers have no formal connection to our home institution.

Our survey on attitudes and use of the Twitter feed was completed by 61 of 95 of our categorical medicine residents (64% response rate). Of 61 responding residents, 33 (54%) residents reported having a Twitter account. However, the majority of our residents (36/61, 59%) reported using Facebook or our website as their preferred way of accessing our Twitter feed. Most residents (47/58, 81%) reported reading our tweets at least once per week, but only 11% (7/61) of our residents accessed the feed daily. The majority of our respondents (42/61, 69%) agreed that our Twitter feed enhanced their education in residency.

Tweets about our morning report were well received with 84% (51/61) of our residents reporting morning report tweets as occasionally or often informative. Most residents (43/61, 70%) agreed that our morning tweets captured the major teaching points. Interestingly, our tweets about medical news and the latest literature garnered greater interest, with 87% (53/61) of our residents finding these tweets informative.

Additional survey data on our intervention is found in Table 2.

**Table 2:** Survey of categorical medicine residents regarding Twitter use and its roles in advancing the educational mission (n=61).

Survey item			n (%)
Reported having personal Twitter account (number of respondents)			33 (54)
**Twitter feed's contribution to educational mission**			
	**Role of Twitter (number in agreement)**		
		Helps residents remember teaching points from the morning report	39 (64)
		Captures the major teaching points from the morning report	43 (70)
		Makes residents feel less removed from the residency program during external rotations (off-campus hospital, emergency ward, etc)	38 (62)
		Enhances overall education in residency	42 (69)
	**Category of tweets (number that rated tweets as informative)**		
		Morning report	51 (84)
		General medical information	53 (87)
		Grand rounds	44 (72)
		Residents' accomplishments, social events, and other institution-specific information	49 (80)

## Discussion

### Principal Findings

Maintaining a residency-run Twitter feed appears to have educational value both internally and externally. Our survey data demonstrates that Twitter was a frequently accessed and appreciated educational resource for residents. Moreover, our robust following outside of our home institution suggests a greater need for resident-directed educational resources in social media.

Though maintaining a Twitter feed requires an additional investment of a chief resident’s time, we believe social media’s strengths in reaching learners justify the effort. Although the educational cornerstone of our residency program has been live teaching conferences, residents have competing priorities that limit attendance. For example, in 2013-2014, our average resident was present for only 48% of conferences they were eligible to attend (excluding post-call days, clinic days, and days off). Giving residents an alternate means of receiving educational material thus represents an important service.

We opted for voluntary participation in our intervention for at least two practical reasons. Most importantly, we wanted to respect residents’ limited time. Additionally, we wanted to use Twitter as an adjunct to—and not a substitute for—our more traditional educational offerings. We felt that compulsory participation could potentially detract from participation in other valuable and mandatory curricula including live conferences and Web-based learning modules.

Our work has some important limitations. Most notably, this intervention occurred at a single site in a single residency program; thus, our results may not be generalizable to all types of medical education settings. Additionally, we did not directly compare Twitter to other educational interventions and thus cannot comment on social media’s relative value in supporting our program’s curricula. Our survey data also relies on self-report of frequency of use rather than actual quantification of use. Finally, we did not directly examine Twitter’s ability to promote knowledge acquisition and retention.

### Professionalism and Legal Concerns

Concerns about professional boundaries and patient privacy might deter residency programs from engaging in social media. Fortunately, guidelines on professional use of social media can be found in a position paper by the American College of Physicians and the Federation of the American Colleges [5], and other tools for understanding professionalism issues are available in the Association of American Medical Colleges' (AAMC) digital literacy toolkit [6]. Many institutions also have their own social media policy. Generally, such guidelines emphasize the sanctity of patient privacy. Experts recommend a “pause before posting” approach when using social media; a brief reflection on a message’s appropriateness often serves as an adequate checkpoint. These general rules of engagement provide an excellent framework for managing a residency program feed.

As program leaders, chief residents are well positioned to role model the use of social media and understand the responsibility and care required to manage a medium prone to misconduct. For example, while we occasionally posted interesting images from case conferences or teaching services, all images were completely deidentified and posted asynchronously to the patient’s hospitalization. Likewise, when tweeting educational materials and original articles from journals, we maintained compliance with fair use by sharing only small parts of published works.

### Future Work

To develop a better understanding of the impact of our program’s Twitter account, we would like to expand the use of data analytics in the coming academic years. Tools embedded in Twitter or offered by third parties allow for meaningful data extraction including the times of day that followers interface with our account and the popularity of specific tweets. These data may better inform future programs on how to best use social media to reach residents.

Analysis of tweets by hashtags would provide additional insight on the content and potential uses of Twitter in resident education. Hashtags likely represent the easiest way to standardize tweets across residency programs. Though other authors have recently analyzed the use of academic conference-specific hashtags on Twitter [7,8], we are not aware of any analysis of hashtags used for regular resident teaching conferences such as the internal medicine #AMReport. Widespread adoption of specific hashtags like #AMReport would create a standardized tweet format and would facilitate large-scale data collection across multiple programs.

### Conclusions

Although recent systematic reviews have relied on low-level outcomes to assess social media’s role in medical education and higher education [9], we believe this medium represents a major frontier in education. We encourage other residency programs to invest the time to maintain a Twitter feed. As our data show, a program-run Twitter account can serve as a meaningful educational tool for the millennial resident.

## Abbreviations

AAMC: Association of American Medical Colleges
CT: computerized tomography
HIPAA: Health Insurance Portability and Accountability Act
IRB: Institutional Review Board
